# Deep sequencing for *de novo* construction of a marine fish *(Sparus aurata)* transcriptome database with a large coverage of protein-coding transcripts

**DOI:** 10.1186/1471-2164-14-178

**Published:** 2013-03-15

**Authors:** Josep A Calduch-Giner, Azucena Bermejo-Nogales, Laura Benedito-Palos, Itziar Estensoro, Gabriel Ballester-Lozano, Ariadna Sitjà-Bobadilla, Jaume Pérez-Sánchez

**Affiliations:** 1 Nutrigenomics and Fish Growth Endocrinology Group, Department of Marine Species Biology, Culture and Pathology, Institute of Aquaculture Torre de la Sal, Castellón, CSIC, Spain; 2 Fish Pathology Group, Department of Marine Species Biology, Culture and Pathology. Institute of Aquaculture Torre de la Sal, Castellón, CSIC, Spain

**Keywords:** *Sparus aurata*, Next-generation sequencing, *De novo* assembly, Transcriptome, Database

## Abstract

**Background:**

The gilthead sea bream (*Sparus aurata*) is the main fish species cultured in the Mediterranean area and constitutes an interesting model of research. Nevertheless, transcriptomic and genomic data are still scarce for this highly valuable species. A transcriptome database was constructed by *de novo* assembly of gilthead sea bream sequences derived from public repositories of mRNA and collections of expressed sequence tags together with new high-quality reads from five cDNA 454 normalized libraries of skeletal muscle (1), intestine (1), head kidney (2) and blood (1).

**Results:**

Sequencing of the new 454 normalized libraries produced 2,945,914 high-quality reads and the *de novo* global assembly yielded 125,263 unique sequences with an average length of 727 nt. Blast analysis directed to protein and nucleotide databases annotated 63,880 sequences encoding for 21,384 gene descriptions, that were curated for redundancies and frameshifting at the homopolymer regions of open reading frames, and hosted at http://www.nutrigroup-iats.org/seabreamdb. Among the annotated gene descriptions, 16,177 were mapped in the Ingenuity Pathway Analysis (IPA) database, and 10,899 were eligible for functional analysis with a representation in 341 out of 372 IPA canonical pathways. The high representation of randomly selected stickleback transcripts by Blast search in the nucleotide gilthead sea bream database evidenced its high coverage of protein-coding transcripts.

**Conclusions:**

The newly assembled gilthead sea bream transcriptome represents a progress in genomic resources for this species, as it probably contains more than 75% of actively transcribed genes, constituting a valuable tool to assist studies on functional genomics and future genome projects.

## Background

The gilthead sea bream (*Sparus aurata*) is a member of the Sparidae family widely and successfully cultured in the Mediterranean region. During the last decade more than 1,200 scientific papers have focused on gilthead sea bream nutrition, immune response, physiology and genetics. This high valuable fish for aquaculture industry becomes, thereby, an interesting animal model, and the development of molecular and genomic tools for research is highly desirable. Previous attempts have been made in this way and the assembly and annotation of more than 40,000 expressed sequence tags (ESTs) allowed the development of specific gilthead sea bream microarrays that have been used in transcriptomic studies of crowding stress [1] and parasite and nutritional challenges [2,3]. Microarray approaches have also been done in this species to assess transcriptome differences between two early larval stages [4], to underline the liver transcriptomic response after cortisol administration [5,6] or to identify the key genes involved in the re-epithelialization process after scale removal [7]. Nevertheless, genomic tools still remain relatively scarce in gilthead sea bream and need to be further improved in comparison to other cultured fish, such as Atlantic salmon (*Salmo salar*) [8], common carp (*Cyprinus carpio*) [9], Nile tilapia (*Oreochromis niloticus*) [10] or turbot (*Scophthalmus maximus*) [11], for which large ESTs collections generated by Sanger sequencing of cDNA libraries are currently available.

With the advent of the next generation sequencing technologies, the gathering of large amounts of sequence data for a given organism at affordable costs is more feasible [12], and high-throughput 454 pyrosequencing has been used to explore the transcriptome of rainbow trout (*Oncorhynchus mykiss*) [13,14], Atlantic cod (*Gadus morhua*) [15] and turbot [16]. In the case of gilthead sea bream, two deep sequencing studies have been reported from whole larval tissues [17] and skeletal muscle [18], yielding 68,289 and 43,461 assembled sequences, respectively. Nevertheless, the assembly of high-throughput sequences from one unique tissue usually results in relatively short sequences. As the annotation success of a sequencing project is highly dependent on the size of the assembled sequences, approaches conducted to obtain longer sequences become desirable. At this respect, it has been proved in some animal models, including rainbow trout [13,14], that the use of two or more tissues for sequencing and assembly increases the number of annotated genes. On this basis, the primary goal of the present study was to generate a large amount of gilthead sea bream transcriptomic reads from metabolically and immunologically relevant tissues by means of the construction of five 454 pyrosequencing libraries, combining them with Sanger sequences from public repositories and our own published data. It comprised nine previously constructed suppression subtractive hybridization (SSH) libraries that were obtained from a variety of tissues (liver, gills, brain, intestine, head kidney, adipose tissue) from animals exposed to confinement stress, parasite infection or a nutritional stress (essential fatty acid deficiencies) [1-3]. Having this into account, tissues selected for the improvement of the current knowledge on gilthead sea bream transcriptome by means of high-throughput sequencing were related to animal growth (skeletal muscle), nutrient digestion (intestine) and immune response (head kidney at two stages of parasitic infection). Because of the importance of the development of non-lethal diagnostic methods, blood was also considered for 454 sequencing. The second goal of this work was to build a reliable assembly database, with a high confidence of functional annotation by means of similarity searches, gene ontology (GO) terms and detailed insights on pathway analysis for its use as a practical tool by the scientific community. This will assist gene discovery and studies on functional genomics, as well as future genome projects of this important fish species.

## Results and discussion

**Figure 1:**
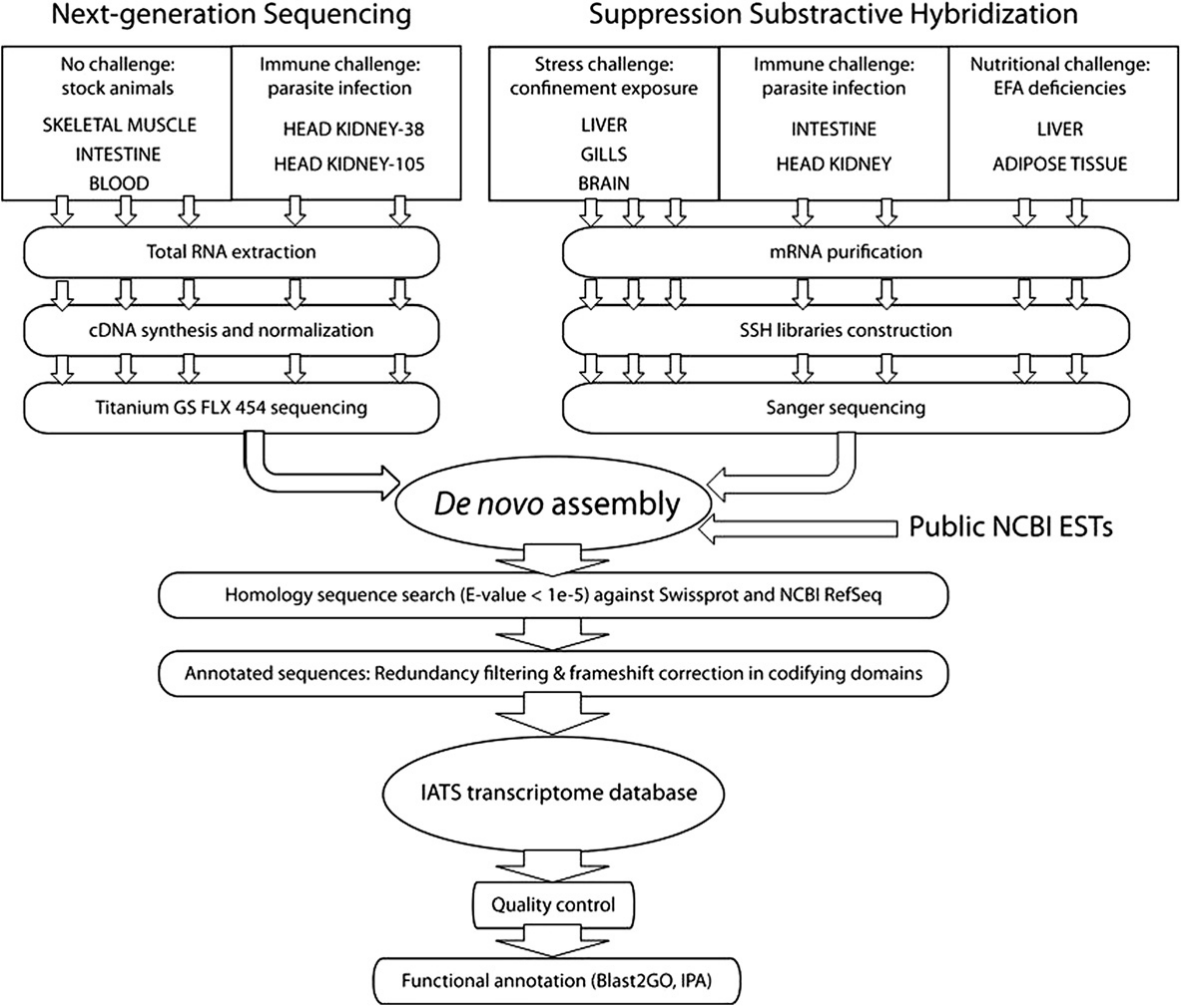
**Schematic representation of the data processing pipeline for the *de novo *assembly and annotation of gilthead sea bream transcriptome.**

Five normalized cDNA libraries from skeletal muscle, intestine, blood and head kidney (short and chronic parasite infection) were constructed and sequenced in separate half-runs using 454-Titanium FLX technology. Table 1 summarizes the results for each one of the five normalized 454 libraries. Collectively, more than 2,900,000 high-quality reads averaging 313 nt each were produced, representing 0.9 Gb of expressed sequence information. For each library, an independent assembly was performed with the Newbler software, and the number of assembled contigs ranged within libraries from 7,800 to 14,008 with an average length of 831–1,148 nt. At this stage and regardless of the tissue library, the number of singletons that failed to assemble remained relatively high. As a result of this, the 67-77% of the information derived from each individual library was supported only by short singleton reads (298–334 nt in length), which is particularly critical given that most assembly software programs include default steps that discard them to give more reliable results, with the unadvised loss of potentially valuable data [19]. This is a complex issue and to solve it, we considered a unique global assembly with the already available mRNA sequences and EST collections in combination with all the unassembled high-quality reads derived from the five 454 libraries (Figure 1).

**Table 1:** Statistics for 454 pyrosequencing libraries

	**Skeletal muscle**	**Intestine**	**Blood**	**HK-38**	**HK-105**
**Pyrosequencing reads**					
High-quality reads	447,166	614,899	576,796	577,112	729,941
Average read length (bp)	294	304	290	349	320
Total Megabases	131.5	186.9	167.3	201.4	233.6
**Assembly statistics**					
Number of contigs	7,808	9,475	12,003	14,008	12,474
Reads assembled	314,628	482,080	389,934	392,195	508,826
Average contig length (bp)	968	1,090	831	1,059	1,148
Number of singletons	48,750	80,527	114,984	105,431	91,885
Average singleton length (nt)	316	298	284	318	334
Total consensus Megabases	23.0	34.3	42.7	48.4	45.0
Average sequences coverage	6.4	6.3	4.0	4.2	5.8

Sequence characteristics of the five 454 normalized libraries derived from skeletal muscle, intestine, blood and head kidney (HK-38, early parasite infection; HK 105, chronic parasite infection).

As a result of the above integrative approach, the average number of reads composing each contig (contig depth) increased from 4.0-6.4 in the individual tissue assemblies to 22.2 in the global assembly (Table 2). This large improvement increased the reliability of the transcriptomic database, yielding 113,927 assembled contigs with an average length of 762 nt that cover the 95.3% of the consensus megabases (86.8 out of 91.1). This finding agrees with the observation that the total number of assembled contigs increased from 43,507 to 50,515 when five skeletal muscle libraries of gilthead sea bream were globally and not individually assembled [18]. Both in that and the present study, the number of 454 reads used for the assembly is of the same order of magnitude (approximately 3 millions), but in our case they are derived from four different tissues and after combination with 82,000 Sanger reads from public repositories the final number of contigs was more than the double (113,927) of that reported in [18].

**Table 2:** ***De novo*assembly statistics**

**Assembly statistics**	
Number of contigs	113,927
Reads assembled	2,765,597
Average contig length (bp)	762
Number of singletons	11,336
Average singleton length (nt)	378
Total consensus Megabases	91.1
Average sequences coverage	22.2

**Figure 2:**
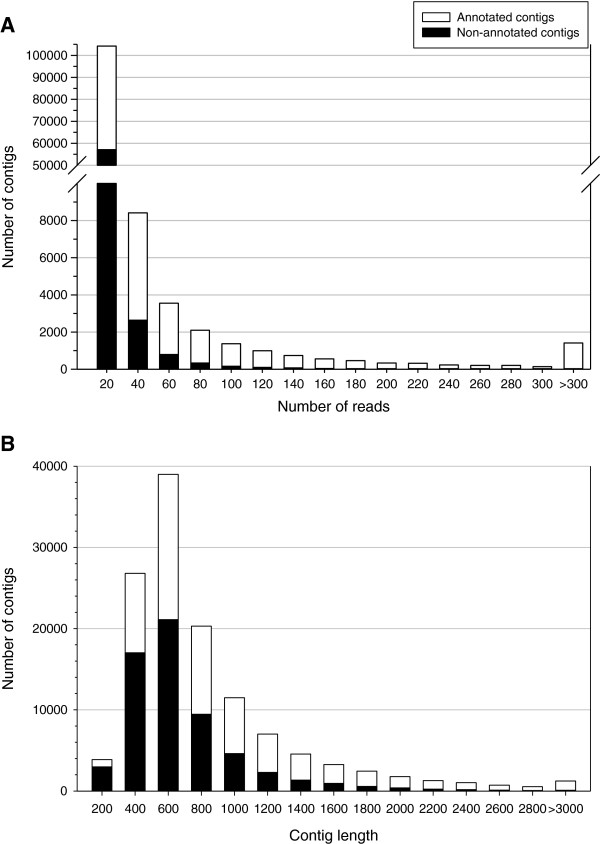
**Characteristics of *de novo *assembled gilthead sea bream transcriptome.** (**A**) Distribution of number of reads per contig. (**B**) Size distribution of reads after the global assembly. The number of annotated (white bars) and non-annotated sequences (black bars) are represented for each category.

Of a total number of 125,263 sequences (contigs and singletons), the 51.0% (63,880 sequences) were annotated using nucleotide and protein reference databases. This annotation ratio is quite higher than those reported in high-throughput sequencing studies conducted in other fish species of interest in aquaculture, such as turbot (44.8%) [16], blunt snout bream (*Megalobrama amblycephala;* 40.5%) [20] or silver carp (*Hypophthalmichthys molitrix;* 26.9%) [21]. This annotation improvement can be explained by the high length (762 nt) of the contigs derived from the present study (Figure 2), which is also inferred when comparisons are made with previous studies in the same species, rendering a contig size of 494 nt and 596 nt in length for 454 libraries of skeletal muscle [18] and whole larvae [17], respectively. The contig depth was increased in parallel and the best BlastX hits for the potentially transcribed proteins yielded 21,384 different Swissprot accessions. This finding suggests that our nucleotide database would contain more than the 75% of the protein coding transcripts of gilthead sea bream, assuming an average size of 25,000-30,000 genes in a fish with a non-recently duplicated genome [22,23].

With independence of a high coverage of protein-coding transcripts, more than 60,000 assemblies still remain without annotation. This relatively high number of unknown sequences could correspond to more divergently evolved genes through vertebrate evolution, though we cannot exclude that some of these sequences may result from assembly artifacts. Alternatively, some of these sequences might correspond to long non-coding RNAs (lncRNAs), which are now emerging as an important class of regulatory transcripts with an extent that increases much more with organism complexity [24,25]. Otherwise, high-throughput sequencing is prone to sequencing errors at homopolymer regions, even when assembled at high coverage, which often give rise to a range of artificial sequences [26-28]. To overcome this issue, an *in silico* correction step was introduced in the pipeline procedure, which allowed to obtain continuous open reading frames for annotated sequences avoiding frameshifting by edition (insertion or deletion) of single nucleotides at homopolymer regions. With this newly developed tool, up to 34% of annotated sequences (21,748 out of 63,880 assembled sequences) were detected to carry one or more frameshifts. Among them, 21,105 were satisfactorily corrected with the pipeline and only 643 needed a manual curation.

**Figure 3:**
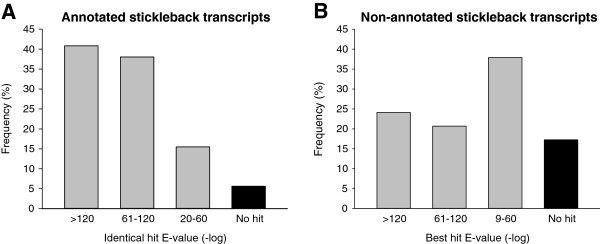
**Quality control of the gilthead sea bream transcriptome annotation.** Frequency distribution of BlastX hit results for annotated (**A**) and non-annotated (**B**) stickleback transcripts.

Quality and reliability of the assembled gilthead sea bream database was assessed by BlastX comparison of 200 randomly chosen transcripts of the stickleback transcriptome. Of note, the 71% of these stickleback transcripts (142 sequences) were originally annotated by the stickleback consortium, and for 94% of them (133 sequences) a positive result with the same annotation was found using our database as a transcriptome reference (Figure 3A). Besides, reliable annotations were found for 48 sequences out of 58 that were not previously annotated in the stickleback transcriptome (Figure 3B). Again this successful result can be explained by the high average length of the assembled gilthead sea bream sequences, obtained by *de novo* global assembly of Sanger sequences and high-throughput sequences of four metabolically and immunologically relevant tissues.

**Figure 4:**
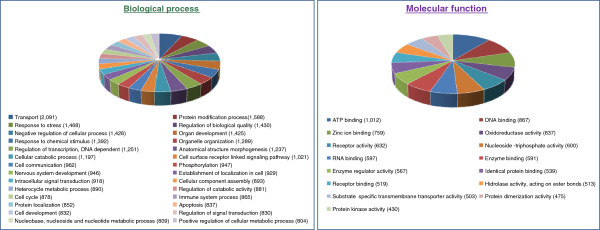
Distribution of multilevel GO annotation terms for biological process and molecular function in the gilthead sea bream transcriptome.

Blast2GO analysis of the different annotations (Figure 4) reveals that the most abundant GO terms related to biological processes were transport (GO:0006810; 2,091 different genes), protein modification process (GO:0036211; 1,558 genes), response to stress (GO:0006950; 1,468 genes), regulation of biological quality (GO:0065008; 1,430 genes) and negative regulation of cellular process (GO:0048523; 1,428 genes). Other highly represented terms are immune system process (GO:0002376; 865 different genes), haemopoiesis (GO:0030097; 289 genes) and coagulation (GO:0050817; 290 genes), which is not surprising given that 3 out of five 454 libraries were derived from blood and head kidney. Regarding skeletal muscle, tissue-specific biological processes like muscle structure development (GO:0061061; 302 genes), muscle cell differentiation (GO:0042692; 200 genes) or muscle contraction (GO:0006936; 156 genes) were also highly represented in the annotated gilthead sea bream transcriptome. Likewise, the intestine participates in nutrient digestion and absorption with also an important role in xenobiotic metabolism [29], and accordingly genes related to the molecular functions hydrolase activity (GO:0016787; 1,821 genes) and oxidoreductase activity (GO:0016491; 637 genes) were highly abundant in our database.

GO terms define gene sets, but the relevance of a given metabolic process or biological pathway in a dataset is determined not only by the number of identified genes, but also by their relative abundance ratio considering the total number of genes involved in each pathway. For this reason, precise mapping of metabolic pathways becomes necessary, and the usefulness of pathway analysis software is largely increasing [30]. To date, the only fish metabolic network available, the public online tool MetaFishNet [31], is still far from completion, so pathway analysis of the gilthead sea bream annotated sequences was performed by means of the Ingenuity Pathway Analysis (IPA) software. Conversion of the annotated gilthead sea bream sequences to IPA elements assumed that the functional classification of genes did not vary too much widely between teleostean fish and model species (human, rat and mouse). In this scenario, from the initial list of 21,384 annotated gilthead sea bream descriptions, the IPA software identified 17,144 Uniprot codes with model species equivalences, and 10,899 of them were eligible for function/pathway analysis. These sequences were represented in 341 IPA canonical pathways mapped from a total of 372, which reinforces the idea that the present gilthead sea bream transcriptome is of high coverage and, thereby, very close to completion of the protein-coding transcriptome. The most representative high-level functional categories are listed in Table 3 with precise information of statistic values and number of represented molecules. The top five canonical pathways were regulation of eIF-4 and p70S6K signaling, purine metabolism, EIF2 signaling, protein ubiquitination pathway, and glucocorticoid receptor signaling. As a corollary, the complete list of canonical pathways with the ratio of gilthead sea bream genes present in each one is provided as Additional file 1, and the graphical representation of representative canonical pathways of processes of interest (mTOR signaling, mitochondrial dysfunction, tight junction signaling, B cell receptor signaling, acute phase response signaling and complement system) are presented in Additional file 2.

**Table 3:** Most significant canonical pathways determined by Ingenuity Pathway Analysis and represented in the assembled gilthead sea bream transcriptome

**Canonical pathway**	**E-value**	**Molecules**	**Canonical pathway**	**E-value**	**Molecules**
Regulation of eIF4 and p70S6K Signaling	1.58E-23	130	Erythropoietin Signaling	8.32E-08	56
Purine Metabolism	7.94E-23	219	Ubiquinone Biosynthesis	9.12E-08	58
EIF2 Signaling	3.98E-21	156	AMPK Signaling	9.33E-08	100
Protein Ubiquitination Pathway	7.94E-20	207	Induction of Apoptosis by HIV1	1.05E-07	51
Glucocorticoid Receptor Signaling	2.51E-17	203	IL-2 Signaling	1,17E-07	46
mTOR Signaling	2.51E-16	148	CD40 Signaling	1,20E-07	53
Molecular Mechanisms of Cancer	6.31E-16	250	Role of NFAT in Cardiac Hypertrophy	1,29E-07	131
Mitochondrial Dysfunction	1.00E-15	114	HGF Signaling	1,48E-07	77
Integrin Signaling	6.31E-14	155	FLT3 Signaling in Hematopoietics	1,55E-07	59
Inositol Phosphate Metabolism	2.51E-13	122	Progenitor Cells		
Thrombin Signaling	1.58E-12	147	Fcγ Receptor-mediated Phagocytosis in	1,58E-07	73
Signaling by Rho Family GTPases	1.58E-12	174	Macrophages and Monocytes		
Estrogen Receptor Signaling	3.16E-12	104	Sphingosine-1-phosphate Signaling	1,66E-07	86
CXCR4 Signaling	5.01E-12	121	FAK Signaling	1,66E-07	69
Oxidative Phosphorylation	6.31E-12	115	Paxillin Signaling	2,09E-07	78
Acute Phase Response Signaling	7.94E-12	130	Small Cell Lung Cancer Signaling	2,40E-07	58
Breast Cancer Regulation by Stathmin1	1.26E-11	147	T Cell Receptor Signaling	3,31E-07	75
Type II Diabetes Mellitus Signaling	3.98E-11	94	B Cell Receptor Signaling	3,31E-07	105
Huntington’s Disease Signaling	5.01E-11	161	Production of Nitric Oxide and Reactive	3,72E-07	126
Pyrimidine Metabolism	7.94E-11	111	Oxygen Species in Macrophages		
NGF Signaling	7.94E-11	86	LPS-stimulated MAPK Signaling	3,89E-07	59
PI3K/AKT Signaling	1.10E-10	96	Insulin Receptor Signaling	3,98E-07	96
RAR Activation	1.38E-10	130	Cyclins and Cell Cycle Regulation	3,98E-07	63
Androgen Signaling	1.62E-10	91	PI3K Signaling in B Lymphocytes	4,57E-07	99
Tight Junction Signaling	1.86E-10	120	IGF-1 Signaling	4,68E-07	76
Chronic Myeloid Leukemia Signaling	4.68E-10	78	p53 Signaling	5,25E-07	72
NRF2-mediated Oxidative Stress Response	8.51E-10	134	VEGF Signaling	5,62E-07	68
PPARα/RXRα Activation	1.12E-09	128	Prostate Cancer Signaling	6,03E-07	64
Actin Cytoskeleton Signaling	1.78E-09	154	Nicotinate and Nicotinamide Metabolism	6,46E-07	77
Ceramide Signaling	2.00E-09	66	Relaxin Signaling	6,46E-07	98
Role of Tissue Factor in Cancer	2.29E-09	87	Glioma Signaling	7,24E-07	73
Hereditary Breast Cancer Signaling	3.31E-09	92	Pentose Phosphate Pathway	7,94E-07	29
ERK/MAPK Signaling	3.72E-09	135	Pancreatic Adenocarcinoma Signaling	8,13E-07	80
NF-κB Activation by Viruses	4.57E-09	62	Mitotic Roles of Polo-Like Kinase	8,51E-07	51
Apoptosis Signaling	4.68E-09	73	Acute Myeloid Leukemia Signaling	9,33E-07	61
Gα12/13 Signaling	4.90E-09	93	PDGF Signaling	9,33E-07	57
Cardiac Hypertrophy Signaling	5.13E-09	159	CNTF Signaling	1,05E-06	44
Rac Signaling	5.37E-09	83	Endometrial Cancer Signaling	1,05E-06	44
Germ Cell-Sertoli Cell Junction Signaling	6.17E-09	118	Thrombopoietin Signaling	1,17E-06	46
ILK Signaling	6.76E-09	135	Melanocyte Development and Pigmentation	1,29E-06	66
Renin-Angiotensin Signaling	7.94E-09	86	Signaling		
Protein Kinase A Signaling	8.51E-09	212	Prolactin Signaling	1,45E-06	58
Aldosterone Signaling in Epithelial Cells	8.91E-09	117	Colorectal Cancer Metastasis Signaling	1,58E-06	161
RhoGDI Signaling	9.12E-09	129	RANK Signaling in Osteoclasts	1,82E-06	67
p70S6K Signaling	1.00E-08	95	IL-17A Signaling in Airway Cells	2,19E-06	51
IL-8 Signaling	1.02E-08	127	Aminoacyl-tRNA Biosynthesis	2,40E-06	30
IL-15 Signaling	1.15E-08	53	G Beta Gamma Signaling	2,51E-06	68
fMLP Signaling in Neutrophils	1.23E-08	85	Telomerase Signaling	2,69E-06	75
Phospholipase C Signaling	1.78E-08	163	IL-6 Signaling	3,16E-06	71
PAK Signaling	1.91E-08	72	Role of BRCA1 in DNA Damage Response	3,24E-06	46
P2Y Purigenic Receptor Signaling Pathway	2.24E-08	93	Death Receptor Signaling	3,39E-06	48
HMGB1 Signaling	3.02E-08	75	IL-3 Signaling	3,39E-06	56
Leukocyte Extravasation Signaling	3.16E-08	137	Starch and Sucrose Metabolism	3,47E-06	54
RhoA Signaling	3.89E-08	86	GM-CSF Signaling	3,47E-06	50
PTEN Signaling	4.07E-08	84	Angiopoietin Signaling	3,47E-06	52
Virus Entry via Endocytic Pathways	4.57E-08	72	Non-Small Cell Lung Cancer Signaling	3,47E-06	52
Growth Hormone Signaling	5.13E-08	57	Role of NFAT in Regulation of the Immune	3,72E-06	120
JAK/Stat Signaling	6.31E-08	52	Response		
Lymphotoxin β Receptor Signaling	7.08E-08	47	Neuregulin Signaling	3,72E-06	67

In order to construct a reliable database of protein-coding transcripts with a minimum of frameshift errors or redundancies, a strict filtering was applied to the annotated sequences after the *in silico* homopolymer correction step. Sequences with a significant hit against Swissprot were selected, and then sequences encoding for a same annotation regardless of the species were clustered. Filtering selected for each annotation non-overlaping sequences and putative complete open reading frames, that made a total of 17,809 sequences that were uploaded in our nucleotide database (http://www.nutrigroup-iats.org/seabreamdb). This online resource is intended to become a dynamic and useful tool for scientific community. With this interface, data can be queried using different strategies, such as several Blast options or direct word search for annotation or GO terms. Search results provide additional information for each sequence, like its length, depth or the related Swissprot accession for the assigned annotation, among others.

## Conclusions

A gilthead sea bream transcriptome database has been constructed by *de novo* assembly of five normalized 454 libraries from metabolically and immunologically relevant tissues combined with public sequences that included nine SSH libraries. This approach yielded 125,263 different sequences, and for 63,880 a reliable annotation was found, resulting in 21,384 different gene descriptions that comprised a vast array of functional categories and biological pathways. This constitutes the largest and most complete transcriptome reported to date for gilthead sea bream, having a size and depth equivalent to those reported in the Ensembl genome database for Atlantic cod and other cultured fish species. The information of annotated contigs has been semi-automatically corrected and filtered for redundancies, and is stored in a web database (http://www.nutrigroup-iats.org/seabreamdb) that has been provided with Blast and other search options for the scientific community. This represents a valuable tool to assist fish phenotyping and the concomitant development of molecular biomarker panels (microarrays or process-focused PCR-arrays) of prognostic and diagnostic value to cope with developmental-, nutritional-, environmental- and disease-related stressors.

## Methods

### Experimental setup and tissue sampling

Juveniles of gilthead sea bream were maintained under intensive rearing conditions in the indoor experimental facilities of the Institute of Aquaculture Torre de la Sal (IATS), following standard conditions of photoperiod and temperature at our latitude (40º5^′^N, 0º10^′^E). Animals were fed with conventional diets and culture densities remained lower than 15 Kg/m^3^. Naïve stock fish were sampled after overnight fasting for blood, skeletal muscle and intestine. Blood was taken from the caudal vein with EDTA-treated syringes and 150 μl were disposed in cooled eppendorf tubes with 500 μl of lysis solution until RNA extraction (Real Total RNA Spin Blood Kit, Durviz). Prior to tissue collection, fish were killed by cervical section. Skeletal muscle and intestine samples were then rapidly excised, frozen in liquid nitrogen and stored at −80°C until RNA extraction. Additionally, another stock of fish was infected with the myxosporean parasite *Enteromyxum leei* by exposure to contaminated effluent, following the infection procedure previously described [32], and samples of head kidney, the equivalent to mammalian bone marrow, were taken after 38 (HK-38, early immune response) and 105 days (HK-105, chronic immune response) post exposure. All procedures were carried out according to the national and institutional regulations on animal experimental handling (IATS-CSIC Review Board).

### RNA extraction

Total RNA from each individual tissue sample was isolated by means of the Ambion MagMax-96 for Microarray kit (Applied Biosystems) after tissue homogenization in TRI reagent at a concentration of 100 mg/ml following the manufacturers’ instructions. Purification of total RNA from fish blood was performed according to the procedure of Real Total RNA Spin Blood Kit (Durviz). RNA quantity and purity was determined by Nanodrop (Thermo Scientific) and Agilent 2100 bioanalyzer (Agilent Technologies). Tissue samples of higher quality (absorbance ratios at 260 nm/280 nm above 1.9 and RNA integrity numbers between 9.2 and 10) were selected for high-throughput sequencing.

### cDNA synthesis and normalization

For each tissue and condition (skeletal muscle, intestine, blood, HK-38, HK-105), a single RNA sample (700 ng) was taken for polyA cDNA synthesis using the MINT kit (Evrogen). To increase the rate recovery of rare and unique transcripts, amplified cDNAs were normalized by duplex-specific nuclease with the Trimmer kit (Evrogen) [33] following manufacturer’s indications. Normalized cDNAs samples were measured with Quant-iT PicoGreen dsDNA quantification Kit (Life Technologies) using a VersaFluorTM Fluorometer system (Bio-Rad).

### Libraries construction, pyrosequencing and assembly

For each normalized cDNA, 500 ng were sheared into small fragments (250–600 nt) by nebulization with compressed nitrogen. Then, sequencing adapters were ligated to the blunt ends of fragments, and an emulsion PCR (emPCR) was performed. After emPCR, beads with the cloned amplicons were enriched, loaded onto the 454 microtiter plate and sequenced with a Titanium GS FLX 454 platform (Roche). The sff files containing all reads for each library have been deposited to NCBI Short Read Archive under accession ERP001436.

The quality of the reads was assessed with PERL scripts developed at Lifesequencing S.L. (Valencia, Spain) for trimming of adaptors and validation of high quality sequences. Only high-quality reads (q-value > 25) passed the filter for further assembly and they were assembled using Newbler version 2.5.3 program (454 Life Science-Roche) with the parameters by default with the cDNA option.

### *De novo* assembly and annotation

According to the presented scheme pipeline (Figure 1), the set of nucleotide sequences for *de novo* gilthead sea bream transcriptome assembly was composed of i) unassembled high-quality reads from five 454 libraries (2,945.914 reads), ii) available mRNA sequences from GenBank database (1,733 sequences) and iii) EST collections made available by the Consortium of Marine Genomics Europe [34] and the AQUAFIRST [1] and AQUAMAX [3] EU projects (80,956 ESTs). All sequences were edited to remove vector and adaptor sequences, and cleaned and filtered before assembly and annotation by the SIGENAE information system (INRA Toulouse, France). Cleaning involved masking of poor quality bases and low complexity sequences such as polyA tails. Filtering removed contaminating sequences (bacteria, yeast) and only high quality sequences with more than 100 bases in length were retained. The global assembly was performed by means of the MIRA software version 3.2.0 [35,36].

Assembled contigs and singletons were annotated searching sequence homologies against following databases: UniProtKB/Swiss-Prot, UniProtKB/TrEMBL, RefSeq Protein, Pfam, RefSeq RNA Index Blast, TIGR Fugu, TIGR Medaka, TIGR Salmon, TIGR Trout, TIGR ZebraFish, UniGene FatheadMinnow, UniGene Fugu, UniGene Human, UniGene KilliFish, UniGene Medaka, UniGene Salmon, UniGene Trout, UniGene ZebraFish, Ensembl Fugu transcripts, Ensembl Human transcripts, Ensembl Medaka transcripts, Ensembl Tetraodon transcripts and Ensembl ZebraFish transcripts. The e-value threshold value to determine similarities was set to 1e-5, and the Uniprot entry to which they received the highest similarity was usually assigned as the gene identity.

### Database quality control

Transcriptome sequences of the fish three-spined stickleback (*Gasterosteus aculeatus*) were retrieved from the Ensembl genome database (http://www.ensembl.org/Gasterosteus_aculeatus). Then, randomly selected stickleback sequences (n=200) were compared in a similarity search by BlastX (E-value < 1e-9) into the newly developed gilthead sea bream assembled transcriptome database. For annotated stickleback transcripts, hit results were only considered positive when the most similar gilthead sea bream sequence shared the same annotation.

### Automated frameshift correction and redundancy filtering

To correct the 454 sequencing errors due to frameshifting at homopolymer regions [28], a pipeline based on the combination of the NCBI-Blast package [37] with HMMER [http://hmmer.janelia.org], ClustalW [38], HMM-FRAME [39], PFAM [40] and GyDB [41] databases of HMMs was designed. Additional file 3 implements a most extensive detail of this pipeline and the corrections (punctual insertions/deletions) performed on each sequence.

GPRO 1.1 software [42] was employed to select the most representative sequences among the distinct annotations for a transcribed gene using the algorithm 1, which is a normalized combination of the most relevant Blast statistics with the assembly depth of the sequences:

$$\begin{array}{l} \text{Algorithm}1=\frac{\text{HSPquery}}{100}\times \frac{\text{HSPhit}}{100}\times \frac{\text{Similarity}}{100}\\ \;\times \frac{1}{E-\text{value}}\times \text{Depth} \end{array}$$

For more extensive information about the statistics composing the algorithm please refer the Blast user guide at NCBI [http://www.ncbi.nlm.nih.gov/books/NBK1763/]. The software reads in csv format the annotation file of the whole transcriptome and then lets to state one or more classificatory filters to run the algorithm. When various sequences shared the same SwissProt description and a nucleotide identity higher than 95%, they were grouped in the same cluster, retaining within the same categorization the sequences covering non-overlapping regions of the mapped Refseq protein or complete sequences sharing the same description.

### Functional analysis

Gene ontology annotation was made from the nucleotide sequences of the most representative contig/singleton for each gene identity by means of the Blast2GO software [43] with a threshold cutoff set at 1e-3. Pathway analysis of annotated sequences was performed using the IPA software (http://www.ingenuity.com). The dynamic canonical pathways contained in IPA are well-characterized metabolic and cell-signaling pathways that come from specific journal articles, review articles, textbooks and the Kyoto encyclopedia of genes and genomes (KEGG). The IPA canonical pathways display genes/proteins involved, their interactions and the cellular and metabolic reactions in which the pathway is involved. To provide analysis, IPA must be supplied with the Uniprot accession of genes belonging to one of the following model species: Human (*Homo sapiens*), house mouse (*Mus musculus*), rat (*Rattus norvegicus*), fruit fly (*Drosophila melanogaster*), thale cress (*Arabidopsis thaliana*), the nematode *Caenorhabditis elegans*, the bacteria *Escherichia coli* or the yeast *Saccharomyces cerevisiae*. Hence, for each annotated sea bream sequence the protein equivalence for one of the three higher vertebrates IPA model species was searched in Uniprot, and the corresponding accession number was included in the analysis.

### Statistics

Fisher’s exact test was used in IPA analysis to estimate the significance of the incidence of different canonical pathways. This method calculates the probability that the association between experimental gene set and the reference gene set associated with a canonical pathway is due to random chance. A P-value ≤ 0.05 was considered statistically significant and indicated a nonrandom enrichment of an experimental dataset by members of a specific pathway.

## Competing interests

The authors declare that they have no competing interests.

## Authors’ contributions

JPS, ASB and JACG conceived and designed the study. IE and ASB performed the parasite challenges. ABN, LBP and GBL maintained and sampled stock fish. JACG and IE performed RNA extractions. JACG and JPS conducted statistical, gene ontology and pathway analysis of data. ABM and LBP contributed to manual curation of the nucleotide database. JACG, ASB and JPS wrote the manuscript. All authors read, critically revised and approved the final manuscript.

## Supplementary Material

Additional file 1 Complete list of IPA canonical pathways, with the ratio of gilthead sea bream sequences of the reported transcriptome detected for each one. Click here for file

Additional file 2 **Biological networks of genes linked to the canonical pathways “mTOR Signaling” (A), “Mitochondrial Dysfunction” (B), “Tight Junction Signaling” (C), “B Cell Receptor Signaling” (D), “Acute phase response signaling” (E) and “Complement System” (F) according to Ingenuity Pathway Analysis.** Genes that are present in the gilthead sea bream nucleotide database are shown with grey shading. Direct interactions are shown as solid lines and indirect as dashed lines. Click here for file

Additional file 3 **Post-processing data and pipeline for frameshift correction.** Zip file contains a Word document with an explanation of the distinct steps of the frameshifts processing pipeline accompanied by a graphical description of the pipeline schematics, and an Excel document reporting the frameshift corrections. Click here for file
