# Supplementary material for: Deep sequencing for de novo construction of a marine fish (Sparus aurata) transcriptome database with a large coverage of protein-coding transcripts
Source: BMC Genomics. 2013 Mar 15;14:178. doi: 10.1186/1471-2164-14-178 (PMC3606596; doi:10.1186/1471-2164-14-178)
Supplement: Additional file 1 — Complete list of IPA canonical pathways, with the ratio of gilthead sea bream sequences of the reported transcriptome detected for each one. [file 1471-2164-14-178-S1.pdf]

Canonical Pathway	E-value	Ratio
14-3-3-mediated Signaling	4.90E-06	0.70
4-1BB Signaling in T Lymphocytes	2.57E-03	0.68
Actin Cytoskeleton Signaling	1.78E-09	0.65
Actin Nucleation by ARP-WASP Complex	1.29E-05	0.68
Activation of IRF by Cytosolic Pattern Recognition Receptors	8.13E-02	0.54
Acute Myeloid Leukemia Signaling	9.33E-07	0.74
Acute Phase Response Signaling	7.94E-12	0.73
Agrin Interactions at Neuromuscular Junction	2.88E-04	0.73
Airway Inflammation in Asthma	---	---
Airway Pathology in Chronic Obstructive Pulmonary Disease	5.62E-03	0.89
Alanine and Aspartate Metabolism	9.55E-04	0.37
Aldosterone Signaling in Epithelial Cells	8.91E-09	0.67
Allograft Rejection Signaling	---	---
Altered T Cell and B Cell Signaling in Rheumatoid Arthritis	---	---
Aminoacyl-tRNA Biosynthesis	2.40E-06	0.40
Aminophosphonate Metabolism	5.25E-02	0.30
Aminosugars Metabolism	1.48E-03	0.41
AMPK Signaling	9.33E-08	0.60
Amyloid Processing	1.10E-04	0.71
Amyotrophic Lateral Sclerosis Signaling	1.55E-02	0.53
Androgen and Estrogen Metabolism	4.48E-01	0.33
Androgen Signaling	1.62E-10	0.63
Angiopoietin Signaling	3.47E-06	0.70
Antigen Presentation Pathway	2.10E-01	0.56
Antiproliferative Role of Somatostatin Receptor 2	8.51E-05	0.68
Antiproliferative Role of TOB in T Cell Signaling	6.17E-04	0.85
Apoptosis Signaling	4.68E-09	0.76
April Mediated Signaling	2.14E-03	0.67
Arginine and Proline Metabolism	1.41E-02	0.27
Aryl Hydrocarbon Receptor Signaling	7.76E-05	0.59
Ascorbate and Aldarate Metabolism	2.40E-01	0.15
Assembly of RNA Polymerase I Complex	1.16E-01	0.54
Assembly of RNA Polymerase II Complex	1.23E-05	0.73
Assembly of RNA Polymerase III Complex	6.46E-02	0.63
Atherosclerosis Signaling	5.24E-01	0.47
ATM Signaling	2.34E-04	0.74
Autoimmune Thyroid Disease Signaling	---	---
Axonal Guidance Signaling	6.61E-05	0.56
B Cell Activating Factor Signaling	9.55E-04	0.69
B Cell Receptor Signaling	3.31E-07	0.67
Basal Cell Carcinoma Signaling	---	---
Bile Acid Biosynthesis	1.91E-01	0.30
Biosynthesis of Steroids	6.17E-02	0.12
Bladder Cancer Signaling	4.27E-01	0.52
BMP signaling pathway	9.77E-03	0.60
Breast Cancer Regulation by Stathmin1	1.26E-11	0.70
Butanoate Metabolism	1.78E-03	0.33
C21-Steroid Hormone Metabolism	---	---
Calcium Signaling	8.71E-04	0.54

Calcium-induced T Lymphocyte Apoptosis	6.17E-02	0.56
cAMP-mediated signaling	3.44E-01	0.53
Cardiac Hypertrophy Signaling	5.13E-09	0.65
Cardiac β-adrenergic Signaling	3.63E-05	0.60
Cardiomyocyte Differentiation via BMP Receptors	---	---
Caveolar-mediated Endocytosis Signaling	5.62E-04	0.62
CCR3 Signaling in Eosinophils	3.47E-05	0.64
CCR5 Signaling in Macrophages	1.12E-02	0.49
CD27 Signaling in Lymphocytes	3.47E-04	0.68
CD28 Signaling in T Helper Cells	1.86E-05	0.63
CD40 Signaling	1.20E-07	0.76
Cdc42 Signaling	2.75E-02	0.54
CDK5 Signaling	5.37E-06	0.70
Cell Cycle Control of Chromosomal Replication	4.47E-02	0.61
Cell Cycle Regulation by BTG Family Proteins	6.76E-04	0.78
Cell Cycle: G1/S Checkpoint Regulation	4.27E-04	0.69
Cell Cycle: G2/M DNA Damage Checkpoint Regulation	1.78E-03	0.67
Cellular Effects of Sildenafil (Viagra)	3.09E-02	0.53
Ceramide Signaling	2.00E-09	0.76
Chemokine Signaling	5.13E-04	0.69
Cholecystokinin/Gastrin-mediated Signaling	5.62E-03	0.62
Chondroitin Sulfate Biosynthesis	---	---
Chronic Myeloid Leukemia Signaling	4.68E-10	0.74
Circadian Rhythm Signaling	6.17E-02	0.60
Citrate Cycle	1.38E-04	0.44
Clathrin-mediated Endocytosis Signaling	5.37E-03	0.59
Cleavage and Polyadenylation of Pre-mRNA	5.13E-03	0.92
CNTF Signaling	1.05E-06	0.80
Coagulation System	2.29E-03	0.71
Colorectal Cancer Metastasis Signaling	1.58E-06	0.62
Complement System	1.74E-05	0.83
Corticotropin Releasing Hormone Signaling	3.63E-02	0.50
CREB Signaling in Neurons	4.90E-06	0.59
Crosstalk between Dendritic Cells and Natural Killer Cells	---	---
CTLA4 Signaling in Cytotoxic T Lymphocytes	1.82E-05	0.69
CXCR4 Signaling	5.01E-12	0.72
Cyanoamino Acid Metabolism	1.37E-01	0.22
Cyclins and Cell Cycle Regulation	3.98E-07	0.71
Cysteine Metabolism	4.21E-01	0.32
Cytotoxic T Lymphocyte-mediated Apoptosis of Target Cells	---	---
D-arginine and D-ornithine Metabolism	5.24E-01	0.06
Death Receptor Signaling	3.39E-06	0.74
Dendritic Cell Maturation	2.28E-01	0.47
D-glutamine and D-glutamate Metabolism	---	---
DNA Double-Strand Break Repair by Homologous Recombination	1.05E-02	0.71
DNA Double-Strand Break Repair by Non-Homologous End Joining	4.27E-02	0.58
DNA Methylation and Transcriptional Repression Signaling	1.05E-02	0.70
Docosahexaenoic Acid (DHA) Signaling	1.41E-03	0.61
Dopamine Receptor Signaling	1.55E-03	0.56
Dopamine-DARPP32 Feedback in cAMP Signaling	2.69E-03	0.56

EGF Signaling	3.98E-06	0.75
Eicosanoid Signaling	2.06E-01	0.44
EIF2 Signaling	3.98E-21	0.78
Endometrial Cancer Signaling	1.05E-06	0.77
Endoplasmic Reticulum Stress Pathway	1.51E-04	0.94
Endothelin-1 Signaling	1.91E-04	0.60
Ephrin Receptor Signaling	7.76E-06	0.60
ERK/MAPK Signaling	3.72E-09	0.66
ERK5 Signaling	3.63E-02	0.63
Erythropoietin Signaling	8.32E-08	0.72
Estrogen Receptor Signaling	3.16E-12	0.77
Estrogen-Dependent Breast Cancer Signaling	9.12E-05	0.66
Extrinsic Prothrombin Activation Pathway	5.75E-02	0.60
Factors Promoting Cardiogenesis in Vertebrates	6.76E-02	0.56
FAK Signaling	1.66E-07	0.68
Fatty Acid Biosynthesis	1.58E-02	0.18
Fatty Acid Elongation in Mitochondria	4.07E-03	0.34
Fatty Acid Metabolism	5.01E-02	0.40
Fc Epsilon RI Signaling	9.55E-05	0.66
Fcγ Receptor-mediated Phagocytosis in Macrophages and Monocytes	1.58E-07	0.72
FcγRIIB Signaling in B Lymphocytes	9.33E-06	0.59
FGF Signaling	1.02E-02	0.61
FLT3 Signaling in Hematopoietic Progenitor Cells	1.55E-07	0.80
fMLP Signaling in Neutrophils	1.23E-08	0.66
Folate Biosynthesis	---	---
Fructose and Mannose Metabolism	1.82E-02	0.25
FXR/RXR Activation	8.91E-03	0.56
G Beta Gamma Signaling	2.51E-06	0.58
G Protein Signaling Mediated by Tubby	3.47E-03	0.62
Galactose Metabolism	8.91E-04	0.32
Gap Junction Signaling	3.09E-05	0.60
Germ Cell-Sertoli Cell Junction Signaling	6.17E-09	0.71
Glioblastoma Multiforme Signaling	2.57E-04	0.60
Glioma Invasiveness Signaling	9.77E-05	0.73
Glioma Signaling	7.24E-07	0.65
Glucocorticoid Receptor Signaling	2.51E-17	0.69
Glutamate Metabolism	2.14E-03	0.38
Glutamate Receptor Signaling	---	---
Glutathione Metabolism	4.45E-01	0.36
Glycerolipid Metabolism	7.94E-02	0.40
Glycerophospholipid Metabolism	7.76E-04	0.45
Glycine, Serine and Threonine Metabolism	8.71E-05	0.37
Glycolysis/Gluconeogenesis	8.91E-04	0.46
Glycosaminoglycan Degradation	6.03E-03	0.37
Glycosphingolipid Biosynthesis - Ganglioseries	1.29E-01	0.30
Glycosphingolipid Biosynthesis - Globoseries	5.13E-01	0.32
Glycosphingolipid Biosynthesis - Lactoseries	---	---
Glycosphingolipid Biosynthesis - Neolactoseries	5.13E-01	0.20
Glyoxylate and Dicarboxylate Metabolism	6.17E-02	0.13
GM-CSF Signaling	3.47E-06	0.75

GNRH Signaling	1.78E-04	0.58
G-Protein Coupled Receptor Signaling	4.72E-01	0.51
Graft-versus-Host Disease Signaling	---	---
Granzyme A Signaling	4.03E-01	0.55
Granzyme B Signaling	5.01E-04	0.94
Growth Hormone Signaling	5.13E-08	0.76
Gα 12/13 Signaling	---	---
Gα12/13 Signaling	4.90E-09	0.73
Hepatic Cholestasis	3.31E-05	0.55
Hepatic Fibrosis / Hepatic Stellate Cell Activation	4.79E-03	0.61
HER-2 Signaling in Breast Cancer	2.88E-05	0.72
Hereditary Breast Cancer Signaling	3.31E-09	0.71
HGF Signaling	1.48E-07	0.73
HIF1α Signaling	2.14E-04	0.67
Histidine Metabolism	4.66E-01	0.23
HMGB1 Signaling	3.02E-08	0.75
Human Embryonic Stem Cell Pluripotency	---	---
Huntington's Disease Signaling	5.01E-11	0.68
Hypoxia Signaling in the Cardiovascular System	1.00E-03	0.71
iCOS-iCOSL Signaling in T Helper Cells	3.98E-04	0.60
IGF-1 Signaling	4.68E-07	0.71
IL-1 Signaling	6.76E-06	0.65
IL-10 Signaling	1.48E-02	0.58
IL-12 Signaling and Production in Macrophages	4.27E-02	0.52
IL-15 Production	3.24E-02	0.63
IL-15 Signaling	1.15E-08	0.79
IL-17 Signaling	4.57E-04	0.70
IL-17A Signaling in Airway Cells	2.19E-06	0.71
IL-17A Signaling in Fibroblasts	6.76E-03	0.65
IL-17A Signaling in Gastric Cells	1.68E-01	0.64
IL-2 Signaling	1.17E-07	0.79
IL-22 Signaling	1.62E-03	0.80
IL-3 Signaling	3.39E-06	0.76
IL-4 Signaling	3.63E-05	0.71
IL-6 Signaling	3.16E-06	0.71
IL-8 Signaling	1.02E-08	0.66
IL-9 Signaling	5.89E-05	0.73
ILK Signaling	6.76E-09	0.70
Induction of Apoptosis by HIV1	1.05E-07	0.77
Inhibition of Angiogenesis by TSP1	9.77E-03	0.64
Inositol Metabolism	1.33E-01	0.28
Inositol Phosphate Metabolism	2.51E-13	0.63
Insulin Receptor Signaling	3.98E-07	0.69
Integrin Signaling	6.31E-14	0.74
Intrinsic Prothrombin Activation Pathway	8.13E-02	0.59
JAK/Stat Signaling	6.31E-08	0.81
Keratan Sulfate Biosynthesis	4.27E-01	0.53
Leptin Signaling in Obesity	4.47E-06	0.69
Leukocyte Extravasation Signaling	3.16E-08	0.69
Lipid Antigen Presentation by CD1	---	---

LPS/IL-1 Mediated Inhibition of RXR Function	2.24E-02	0.54
LPS-stimulated MAPK Signaling	3.89E-07	0.72
LXR/RXR Activation	7.41E-02	0.53
Lymphotoxin β Receptor Signaling	7.08E-08	0.77
Lysine Biosynthesis	5.64E-01	0.09
Lysine Degradation	1.91E-05	0.35
Macropinocytosis Signaling	3.63E-05	0.68
Maturity Onset Diabetes of Young (MODY) Signaling	---	---
Mechanisms of Viral Exit from Host Cells	9.55E-04	0.69
Melanocyte Development and Pigmentation Signaling	1.29E-06	0.72
Melanoma Signaling	5.62E-06	0.78
Melatonin Signaling	5.01E-03	0.61
Methane Metabolism	3.80E-02	0.19
Methionine Metabolism	1.38E-04	0.32
MIF Regulation of Innate Immunity	4.98E-01	0.44
MIF-mediated Glucocorticoid Regulation	---	---
Mismatch Repair in Eukaryotes	3.16E-05	0.67
Mitochondrial Dysfunction	1.00E-15	0.65
Mitotic Roles of Polo-Like Kinase	8.51E-07	0.79
Molecular Mechanisms of Cancer	6.31E-16	0.66
MSP-RON Signaling Pathway	9.55E-02	0.57
mTOR Signaling	2.51E-16	0.74
Myc Mediated Apoptosis Signaling	1.26E-05	0.77
Natural Killer Cell Signaling	2.34E-02	0.59
Neuregulin Signaling	3.72E-06	0.66
Neuropathic Pain Signaling In Dorsal Horn Neurons	3.72E-02	0.57
Neuroprotective Role of THOP1 in Alzheimer's Disease	4.33E-01	0.41
Neurotrophin/TRK Signaling	2.75E-03	0.62
NF-κB Activation by Viruses	4.57E-09	0.76
NF-κB Signaling	1.55E-05	0.65
NGF Signaling	7.94E-11	0.74
N-Glycan Biosynthesis	6.31E-04	0.41
N-Glycan Degradation	4.47E-02	0.70
Nicotinate and Nicotinamide Metabolism	6.46E-07	0.56
Nitric Oxide Signaling in the Cardiovascular System	5.37E-02	0.49
Nitrogen Metabolism	4.16E-01	0.17
Non-Small Cell Lung Cancer Signaling	3.47E-06	0.66
Notch Signaling	3.03E-01	0.51
NRF2-mediated Oxidative Stress Response	8.51E-10	0.70
Nucleotide Excision Repair Pathway	6.03E-06	0.89
Nucleotide Sugars Metabolism	4.68E-02	0.11
Nur77 Signaling in T Lymphocytes	5.35E-01	0.46
O-Glycan Biosynthesis	2.08E-01	0.42
Oncostatin M Signaling	1.02E-05	0.86
One Carbon Pool by Folate	6.17E-02	0.41
Ovarian Cancer Signaling	5.01E-02	0.56
OX40 Signaling Pathway	---	---
Oxidative Phosphorylation	6.31E-12	0.72
P2Y Purigenic Receptor Signaling Pathway	2.24E-08	0.66
p38 MAPK Signaling	1.22E-01	0.59

p53 Signaling	5.25E-07	0.75
p70S6K Signaling	1.00E-08	0.71
PAK Signaling	1.91E-08	0.67
Pancreatic Adenocarcinoma Signaling	8.13E-07	0.67
Pantothenate and CoA Biosynthesis	2.24E-02	0.25
Parkinson's Signaling	4.89E-01	0.56
Paxillin Signaling	2.09E-07	0.70
PDGF Signaling	9.33E-07	0.72
Pentose and Glucuronate Interconversions	3.80E-02	0.20
Pentose Phosphate Pathway	7.94E-07	0.36
Phenylalanine Metabolism	3.03E-01	0.20
Phenylalanine, Tyrosine and Tryptophan Biosynthesis	3.80E-02	0.19
Phospholipase C Signaling	1.78E-08	0.63
Phospholipid Degradation	6.46E-02	0.51
Phototransduction Pathway	---	---
PI3K Signaling in B Lymphocytes	4.57E-07	0.67
PI3K/AKT Signaling	1.10E-10	0.69
PKCθ Signaling in T Lymphocytes	5.75E-05	0.57
Polyamine Regulation in Colon Cancer	4.27E-02	0.55
PPAR Signaling	7.94E-04	0.62
PPARα/RXRα Activation	1.12E-09	0.68
Primary Immunodeficiency Signaling	2.26E-01	0.49
Production of Nitric Oxide and Reactive Oxygen Species in Macrophages	3.72E-07	0.60
Prolactin Signaling	1.45E-06	0.73
Propanoate Metabolism	9.77E-05	0.36
Prostate Cancer Signaling	6.03E-07	0.66
Protein Kinase A Signaling	8.51E-09	0.64
Protein Ubiquitination Pathway	7.94E-20	0.76
PTEN Signaling	4.07E-08	0.68
Purine Metabolism	7.94E-23	0.55
PXR/RXR Activation	6.17E-02	0.48
Pyrimidine Metabolism	7.94E-11	0.51
Pyruvate Metabolism	1.38E-03	0.35
Rac Signaling	5.37E-09	0.68
RAN Signaling	3.16E-05	0.70
RANK Signaling in Osteoclasts	1.82E-06	0.71
RAR Activation	1.38E-10	0.70
Reelin Signaling in Neurons	1.02E-04	0.71
Regulation of Actin-based Motility by Rho	4.57E-05	0.68
Regulation of eIF4 and p70S6K Signaling	1.58E-23	0.77
Regulation of IL-2 Expression in Activated and Anergic T Lymphocytes	1.35E-05	0.67
Relaxin Signaling	6.46E-07	0.62
Renal Cell Carcinoma Signaling	5.13E-06	0.74
Renin-Angiotensin Signaling	7.94E-09	0.68
Retinoic acid Mediated Apoptosis Signaling	---	---
Retinol Metabolism	2.45E-02	0.49
RhoA Signaling	3.89E-08	0.75
RhoGDI Signaling	9.12E-09	0.64
Riboflavin Metabolism	3.25E-01	0.25
Role of BRCA1 in DNA Damage Response	3.24E-06	0.75

Role of CHK Proteins in Cell Cycle Checkpoint Control	1.74E-05	0.83
Role of Hypercytokinemia/hyperchemokinemia in the Pathogenesis of Influenza	---	---
Role of IL-17A in Arthritis	2.29E-03	0.62
Role of IL-17A in Psoriasis	---	---
Role of IL-17F in Allergic Inflammatory Airway Diseases	6.31E-02	0.61
Role of JAK family kinases in IL-6-type Cytokine Signaling	1.91E-04	0.82
Role of JAK1 and JAK3 in γc Cytokine Signaling	7.59E-03	0.63
Role of JAK1, JAK2 and TYK2 in Interferon Signaling	1.15E-01	0.59
Role of JAK2 in Hormone-like Cytokine Signaling	7.76E-02	0.62
Role of Lipids/Lipid Rafts in the Pathogenesis of Influenza	---	---
Role of Macrophages, Fibroblasts and Endothelial Cells in Rheumatoid Arthritis	1.38E-03	0.55
Role of MAPK Signaling in the Pathogenesis of Influenza	4.90E-02	0.61
Role of NANOG in Mammalian Embryonic Stem Cell Pluripotency	1.99E-01	0.54
Role of NFAT in Cardiac Hypertrophy	1.29E-07	0.62
Role of NFAT in Regulation of the Immune Response	3.72E-06	0.60
Role of Oct4 in Mammalian Embryonic Stem Cell Pluripotency	1.91E-01	0.60
Role of Osteoblasts, Osteoclasts and Chondrocytes in Rheumatoid Arthritis	4.07E-02	0.53
Role of Pattern Recognition Receptors in Recognition of Bacteria and Viruses	1.82E-03	0.62
Role of PI3K/AKT Signaling in the Pathogenesis of Influenza	2.72E-01	0.50
Role of PKR in Interferon Induction and Antiviral Response	1.51E-05	0.74
Role of RIG1-like Receptors in Antiviral Innate Immunity	2.30E-01	0.53
Role of Tissue Factor in Cancer	2.29E-09	0.76
Role of Wnt/GSK-3? Signaling in the Pathogenesis of Influenza	---	---
SAPK/JNK Signaling	2.34E-05	0.68
Selenoamino Acid Metabolism	3.89E-02	0.33
Semaphorin Signaling in Neurons	1.00E-02	0.69
Serotonin Receptor Signaling	---	---
Sertoli Cell-Sertoli Cell Junction Signaling	4.47E-06	0.62
Signaling by Rho Family GTPases	1.58E-12	0.68
Small Cell Lung Cancer Signaling	2.40E-07	0.65
Sonic Hedgehog Signaling	1.08E-01	0.58
Sphingolipid Metabolism	1.32E-02	0.48
Sphingosine-1-phosphate Signaling	1.66E-07	0.69
Spliceosomal Cycle	---	---
Starch and Sucrose Metabolism	3.47E-06	0.32
Stilbene, Coumarine and Lignin Biosynthesis	1.62E-02	0.20
Sulfur Metabolism	4.98E-01	0.19
Synaptic Long Term Depression	3.72E-02	0.52
Synaptic Long Term Potentiation	3.72E-02	0.56
Synthesis and Degradation of Ketone Bodies	9.77E-02	0.47
Systemic Lupus Erythematosus Signaling	4.37E-03	0.54
T Cell Receptor Signaling	3.31E-07	0.69
T Helper Cell Differentiation	---	---
Taurine and Hypotaurine Metabolism	4.78E-01	0.19
Telomerase Signaling	2.69E-06	0.73
Telomere Extension by Telomerase	3.72E-01	0.53
TGF-β Signaling	1.32E-02	0.61
Thrombin Signaling	1.58E-12	0.71
Thrombopoietin Signaling	1.17E-06	0.73
Thyroid Cancer Signaling	3.31E-01	0.54

Tight Junction Signaling	1.86E-10	0.73
TNFR1 Signaling	3.02E-05	0.74
TNFR2 Signaling	8.32E-03	0.65
Toll-like Receptor Signaling	2.04E-03	0.66
TR/RXR Activation	3.98E-06	0.68
TREM1 Signaling	1.05E-04	0.64
Tryptophan Metabolism	8.32E-02	0.33
Tumoricidal Function of Hepatic Natural Killer Cells	3.24E-04	0.88
TWEAK Signaling	9.77E-03	0.64
Type I Diabetes Mellitus Signaling	7.08E-04	0.61
Type II Diabetes Mellitus Signaling	3.98E-11	0.59
Tyrosine Metabolism	2.62E-01	0.21
Ubiquinone Biosynthesis	9.12E-08	0.51
Urea Cycle and Metabolism of Amino Groups	6.76E-04	0.33
Valine, Leucine and Isoleucine Biosynthesis	8.91E-03	0.24
Valine, Leucine and Isoleucine Degradation	5.75E-04	0.44
VDR/RXR Activation	5.33E-01	0.51
VEGF Signaling	5.62E-07	0.69
Virus Entry via Endocytic Pathways	4.57E-08	0.72
Wnt/ß-catenin Signaling	---	---
Xenobiotic Metabolism Signaling	7.76E-04	0.59
α-Adrenergic Signaling	3.72E-06	0.63
β-alanine Metabolism	2.14E-02	0.34
