# Supplementary material for: Deep sequencing for de novo construction of a marine fish (Sparus aurata) transcriptome database with a large coverage of protein-coding transcripts
Source: BMC Genomics. 2013 Mar 15;14:178. doi: 10.1186/1471-2164-14-178 (PMC3606596; doi:10.1186/1471-2164-14-178)
Supplement: Additional file 2 — Biological networks of genes linked to the canonical pathways “mTOR Signaling” (A), “Mitochondrial Dysfunction” (B), “Tight Junction Signaling” (C), “B Cell Receptor Signaling” (D), “Acute phase response signaling” (E) and “Complement System” (F) according to Ingenuity Pathway Analysis. Genes that are present in the gilthead sea bream nucleotide database are shown with grey shading. Direct interactions are shown as solid lines and indirect as dashed lines. [file 1471-2164-14-178-S2.pdf]

A

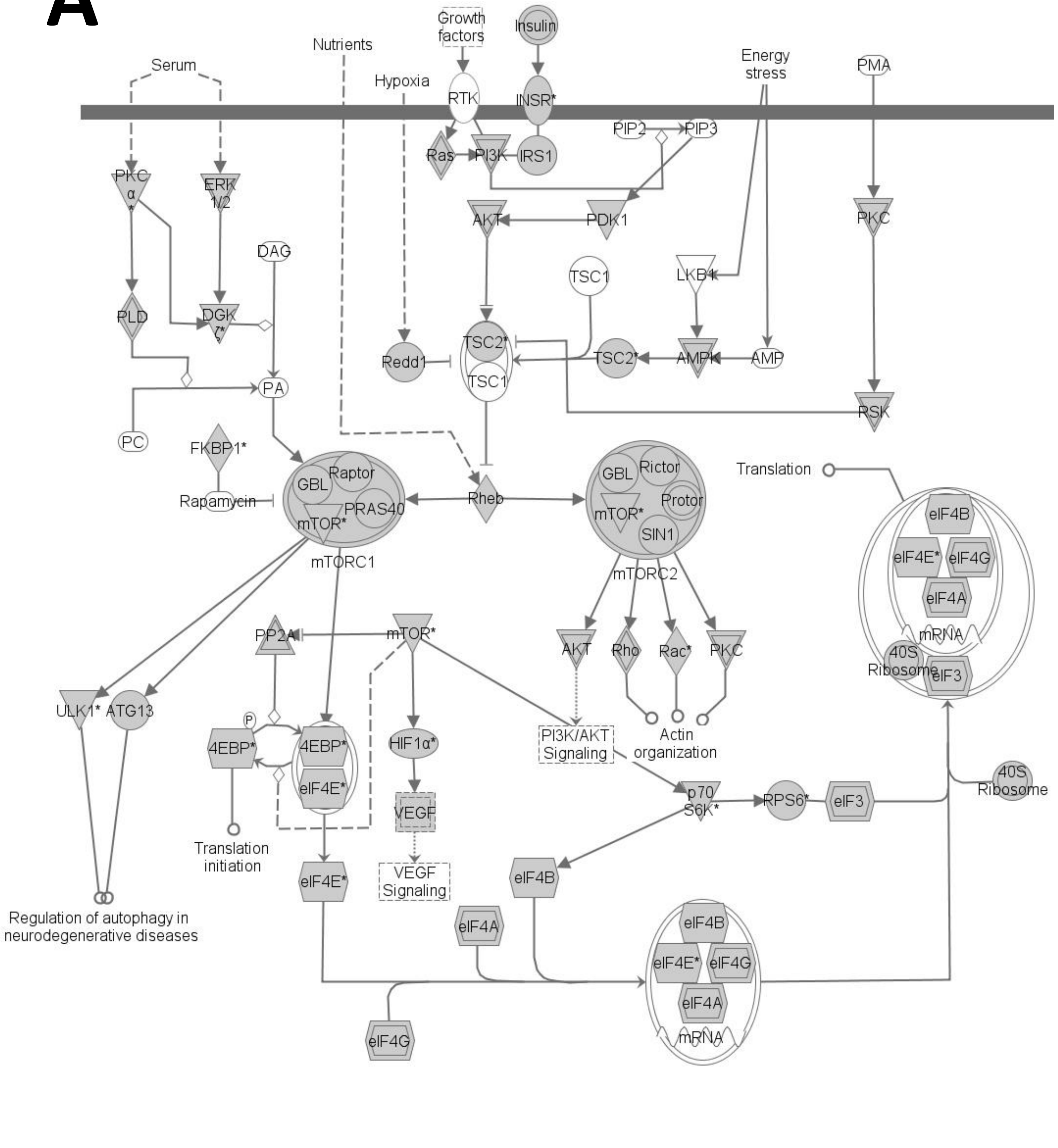

Serum
Nutrients
Growth factors
Insulin
Hypoxia
Energy stress
PMA
RTK
INSR*
PIP2
PIP3
Ras
PI3K
IRS1
PKC α *
ERK 1/2
AKT
PDK1
PKC
DAG
TSC1
LKB1
PLD
DGK ζ*
Redd1
TSC2*
TSC1
TSC2*
AMPK
AMP
PA
RSK
PC
FKBP1*
Rapamycin
GBL
Raptor
PRAS40
mTOR*
mTORC1
Rheb
GBL
Rictor
Protor
mTOR*
SIN1
mTORC2
Translation
eIF4B
eIF4E*
eIF4G
eIF4A
mRNA
40S Ribosome
eIF3
PP2A
mTOR*
AKT
Rho
Rac*
PKC
ULK1*
ATG13
P
4EBP*
4EBP*
eIF4E*
HIF1α*
VEGF
PI3K/AKT Signaling
Actin organization
40S Ribosome
p70 S6K*
RPS6*
eIF3
Translation initiation
eIF4E*
VEGF Signaling
eIF4B
Regulation of autophagy in neurodegenerative diseases
eIF4A
eIF4B
eIF4E*
eIF4G
eIF4A
mRNA
eIF4G

B

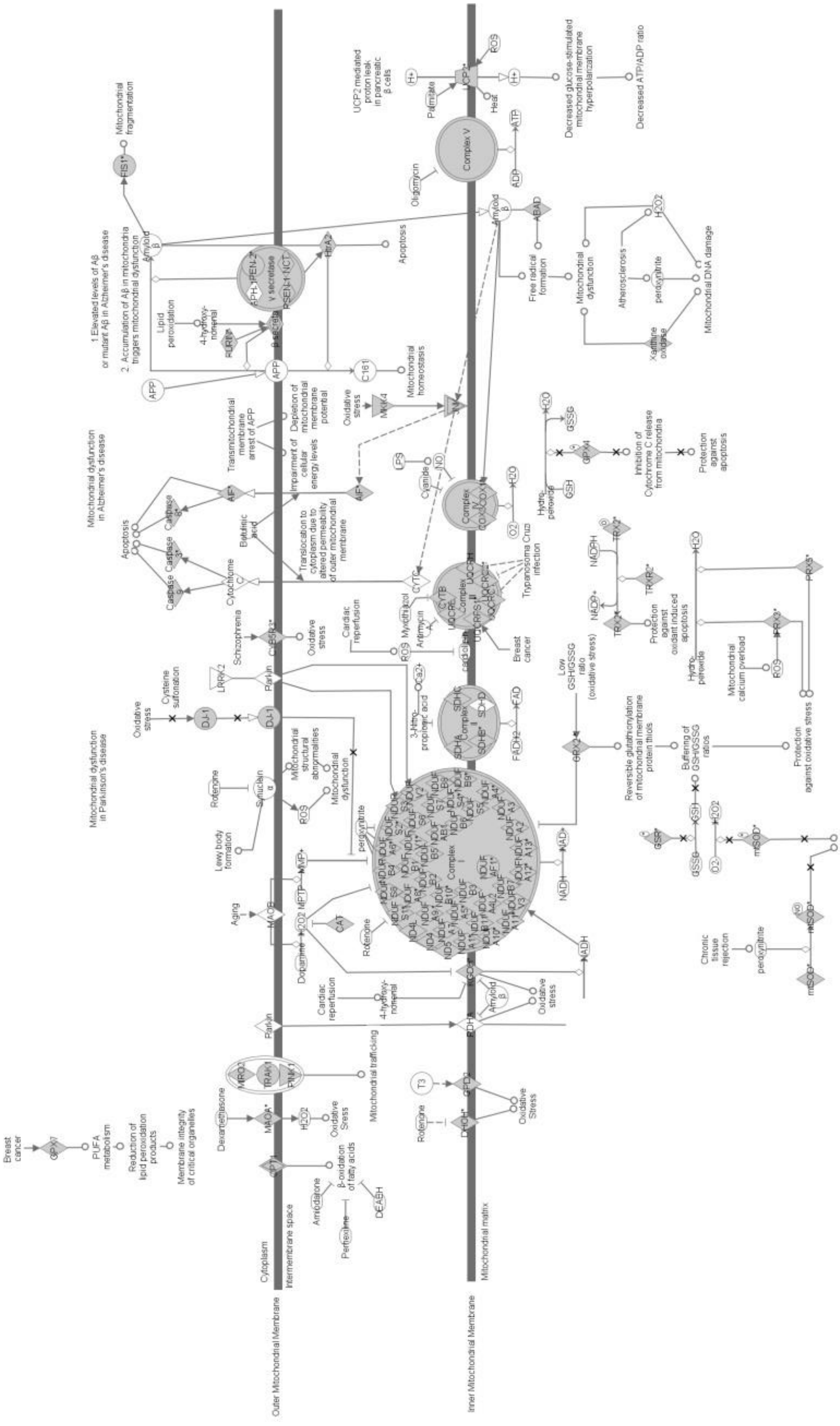
Outer Mitochondrial Membrane
Inner Mitochondrial Membrane
Cytoplasm
Intermembrane space
Mitochondrial matrix
Mitochondrial dysfunction in Parkinson's disease
Mitochondrial dysfunction in Alzheimer's disease
1. Elevated levels of Aβ or mutant Aβ in Alzheimer's disease
2. Accumulation of Aβ in mitochondria triggers mitochondrial dysfunction
Mitochondrial fragmentation
UCP2 mediated proton leak in pancreatic β cells
Decreased glucose-stimulated mitochondrial membrane hyperpolarization
Decreased ATP/ADP ratio
Complex V
Oligomycin
Apoptosis
Mitochondrial homeostasis
Free radical formation
Mitochondrial dysfunction
Atherosclerosis
peroxynitrite
Mitochondrial DNA damage
Xanthine oxidase
Lipid peroxidation
4-hydroxy-nonenal
Transmitochondrial membrane arrest of APP
Depletion of mitochondrial membrane potential
Impairment of cellular energy levels
Oxidative stress
Translocation to cytoplasm due to altered permeability of outer mitochondrial membrane
Botulinic acid
Cytochrome C
Trypanosoma Cruzi infection
Inhibition of Cytochrome C release from mitochondria
Protection against apoptosis
Protection against oxidant induced apoptosis
Mitochondrial calcium overload
Schizophrenia
Cardiac reperfusion
Breast cancer
Cysteine sulfonation
Low GSH/GSSG ratio (oxidative stress)
Reversible glutathionylation of mitochondrial membrane protein thiols
Buffering of GSH/GSSG ratios
Protection against oxidative stress
Mitochondrial structural abnormalities
Lewy body formation
Rotenone
3-Nitro-propionic acid
Protection of FeS cluster containing mitochondrial enzymes
Chronic tissue rejection
Aging
Dopamine
Mitochondrial trafficking
Dexamethasone
PUFA metabolism
Reduction of lipid peroxidation products
Membrane integrity of critical organelles
β-oxidation of fatty acids
Amiodarone
Perhexiline
DEAEH

C

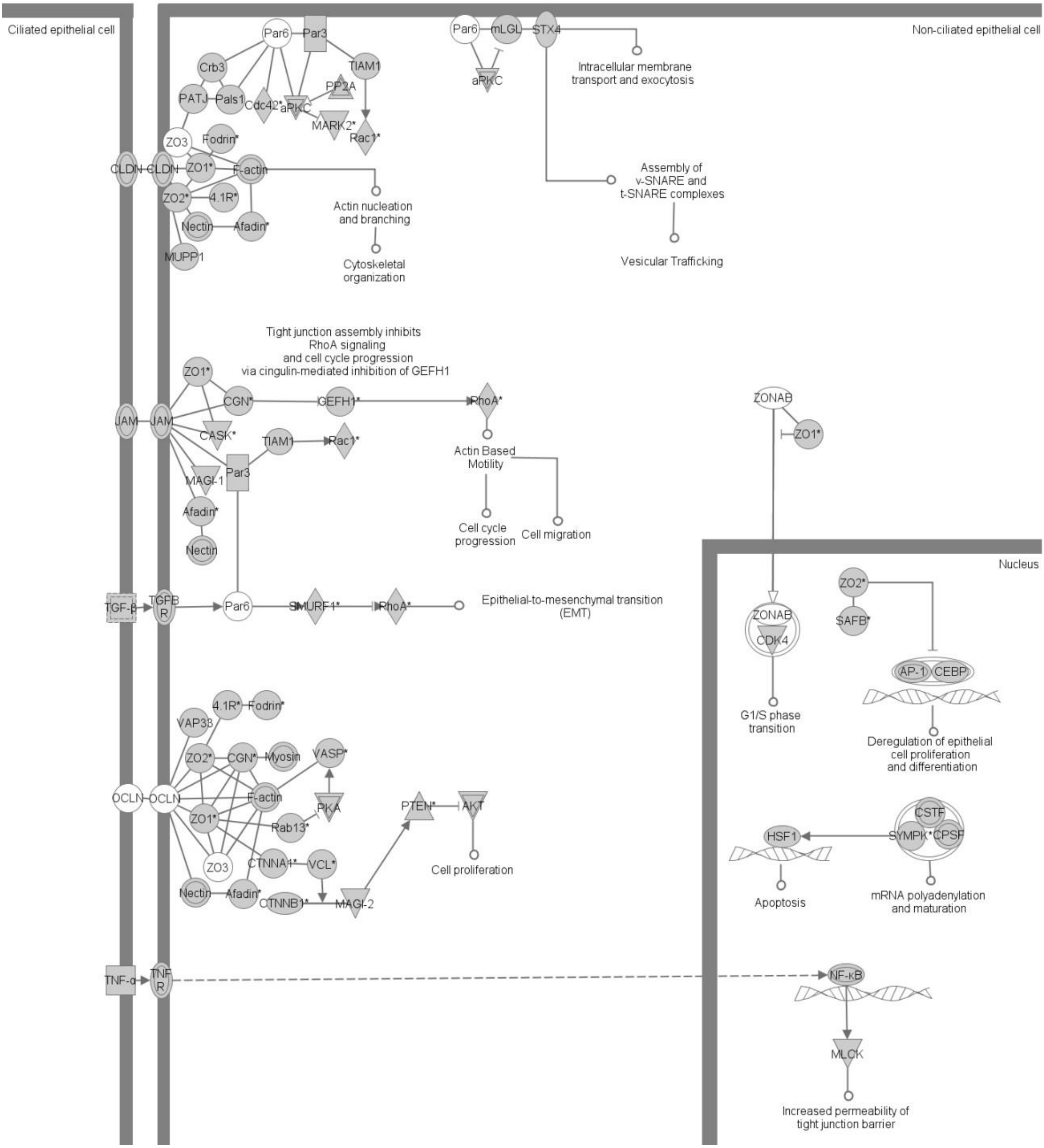

Ciliated epithelial cell
Non-ciliated epithelial cell
Par6
Par3
Crb3
TIAM1
PATJ
Pals1
Cdc42*
aPKC
PP2A
MARK2*
Rac1*
ZO3
Fodrin*
CLDN
CLDN
ZO1*
F-actin
ZO2*
4.1R*
Nectin
Afadin*
MUPP1
Actin nucleation and branching
Cytoskeletal organization
Par6
mLGL
STX4
aPKC
Intracellular membrane transport and exocytosis
Assembly of v-SNARE and t-SNARE complexes
Vesicular Trafficking
Tight junction assembly inhibits RhoA signaling and cell cycle progression via cingulin-mediated inhibition of GEFH1
ZO1*
CGN*
GEFH1*
RhoA*
JAM
JAM
CASK*
TIAM1
Rac1*
MAGI-1
Par3
Afadin*
Nectin
Actin Based Motility
Cell cycle progression
Cell migration
ZONAB
ZO1*
Nucleus
TGF-β
TGFB R
Par6
SMURF1*
RhoA*
Epithelial-to-mesenchymal transition (EMT)
ZONAB
CDK4
G1/S phase transition
ZO2*
SAFB*
AP-1
CEBP
Deregulation of epithelial cell proliferation and differentiation
4.1R*
Fodrin*
VAP33
ZO2*
CGN*
Myosin
VASP*
OCLN
OCLN
F-actin
PKA
PTEN*
AKT
ZO1*
Rab13*
ZO3
CTNNA1*
VCL*
Nectin
Afadin*
CTNNB1*
MAGI-2
Cell proliferation
CSTF
HSF1
SYMPK*
CPSF
Apoptosis
mRNA polyadenylation and maturation
TNF-α
TNF R
NF-κB
MLCK
Increased permeability of tight junction barrier

D

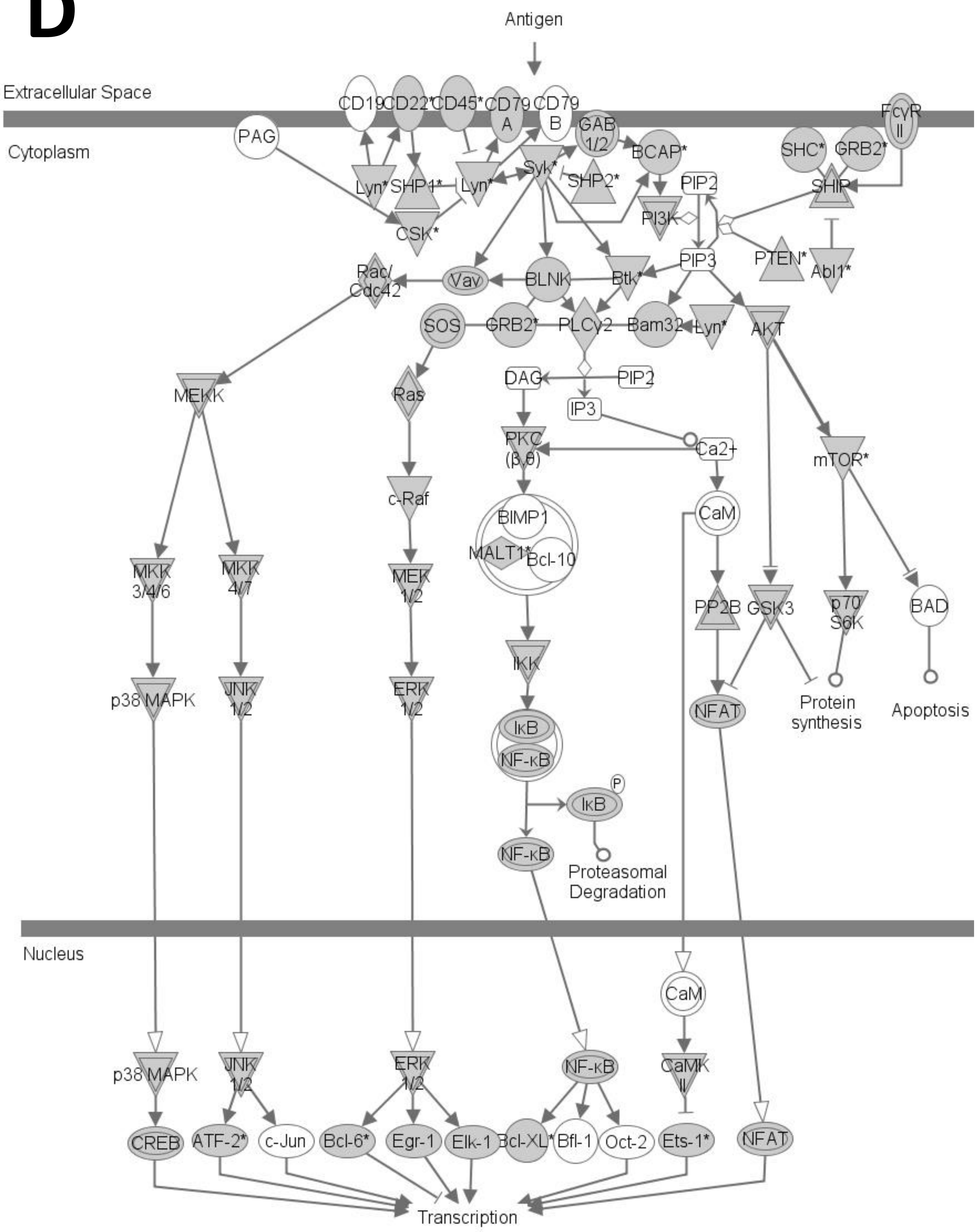
Antigen
Extracellular Space
Cytoplasm
Nucleus
CD19
CD22*
CD45*
CD79 A
CD79 B
GAB 1/2
FcγR II
PAG
Lyn*
SHP1*
Syk*
SHP2*
BCAP*
SHC*
GRB2*
SHIP
CSK*
PIP2
PI3K
PIP3
PTEN*
Abl1*
Rac/ Cdc42*
Vav
BLNK
Btk*
SOS
GRB2*
PLCγ2
Bam32
AKT
MEKK
Ras
DAG
IP3
PKC (β,θ)
Ca2+
mTOR*
c-Raf
BIMP1
MALT1*
Bcl-10
CaM
MKK 3/4/6
MKK 4/7
MEK 1/2
PP2B
GSK3
p70 S6K
BAD
IKK
p38 MAPK
JNK 1/2
ERK 1/2
NFAT
Protein synthesis
Apoptosis
IκB
NF-κB
P
Proteasomal Degradation
CaMK II
CREB
ATF-2*
c-Jun
Bcl-6*
Egr-1
Elk-1
Bcl-XL*
Bfl-1
Oct-2
Ets-1*
Transcription

E

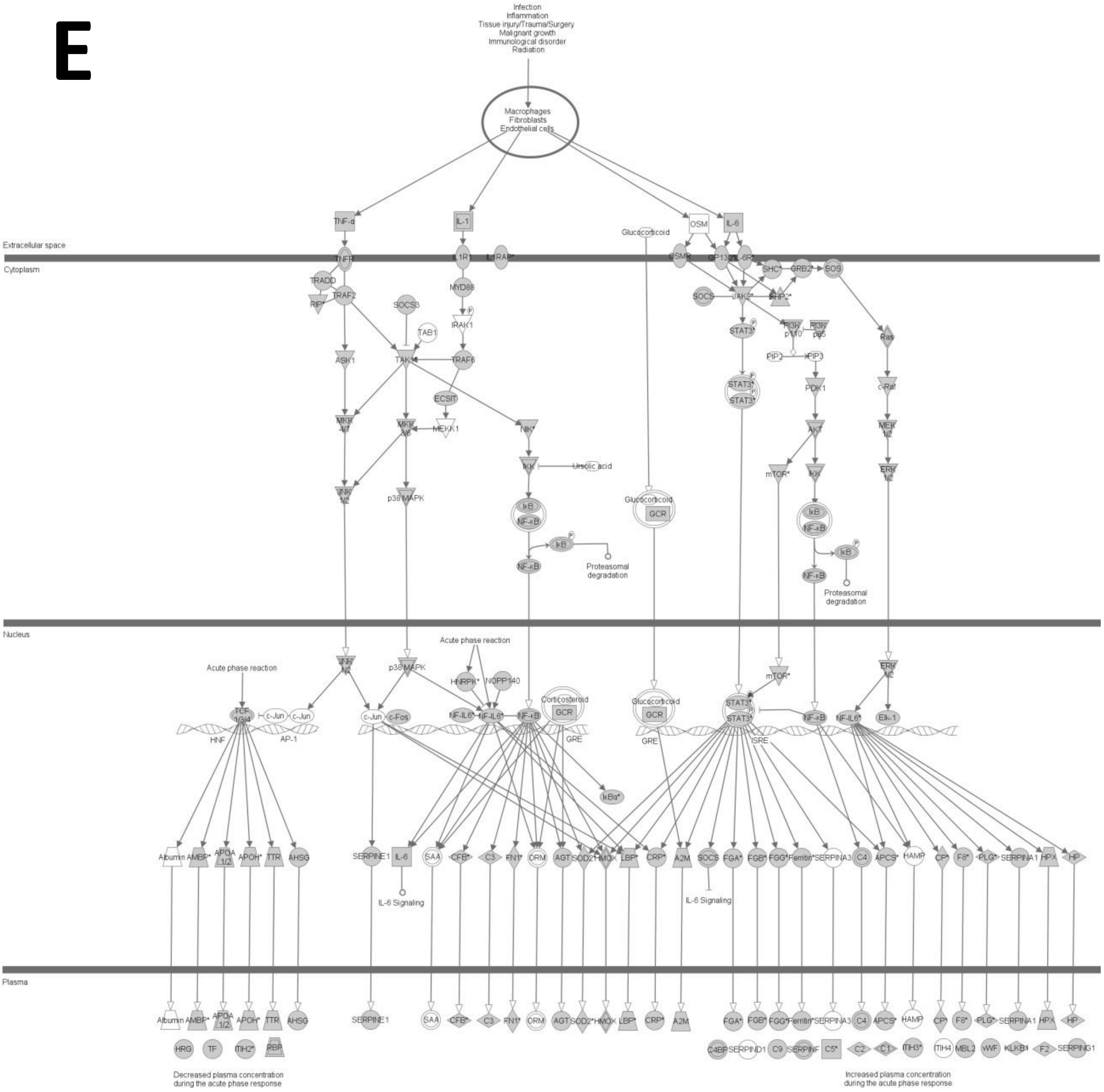

Infection
Inflammation
Tissue injury/Trauma/Surgery
Malignant growth
Immunological disorder
Radiation
Macrophages
Fibroblasts
Endothelial cells
Extracellular space
Cytoplasm
Nucleus
Plasma
TNF-α
IL-1
Glucocorticoid
OSM
IL-6
TNFR
IL1R1
IL1RAP*
OSMR
GP130
IL-6R*
SHC*
GRB2*
SOS
TRADD
TRAF2
RIP*
MYD88
IRAK1
SOCS3
TAB1
SOCS
JAK2*
SHP2*
STAT3*
PI3K p110
PI3K p85
Ras
PIP2
PIP3
ASK1
TAK1
TRAF6
PDK1
c-Raf
ECSIT
MKK 4/7
MKK 3/6
MEKK1
NIK*
AKT
MEK 1/2
IKK
Ursolic acid
mTOR*
ERK 1/2
JNK 1/2
p38 MAPK
IkB
NF-κB
GCR
Proteasomal degradation
Acute phase reaction
HNRPK*
NOPP140
TCF 1/3/4
c-Jun
c-Fos
NF-IL6*
Corticosteroid
Elk-1
HNF
AP-1
GRE
ISRE
IkBα*
Albumin
AMBP*
APOA 1/2
APOH*
TTR
AHSG
SERPINE1
SAA
CFB*
C3
FN1*
ORM
AGT
SOD2*
HMOX
LBP*
CRP*
A2M
FGA*
FGB*
FGG*
Ferritin*
SERPINA3
C4
APCS*
HAMP
CP*
F8*
PLG*
SERPINA1
HPX
HP
IL-6 Signaling
HRG
TF
ITIH2*
RBP
C4BP
SERPIND1
C9
SERPINF
C5*
C2
C1*
ITIH3*
ITIH4
MBL2
vWF
KLKB1
F2
SERPING1
Decreased plasma concentration during the acute phase response
Increased plasma concentration during the acute phase response

F

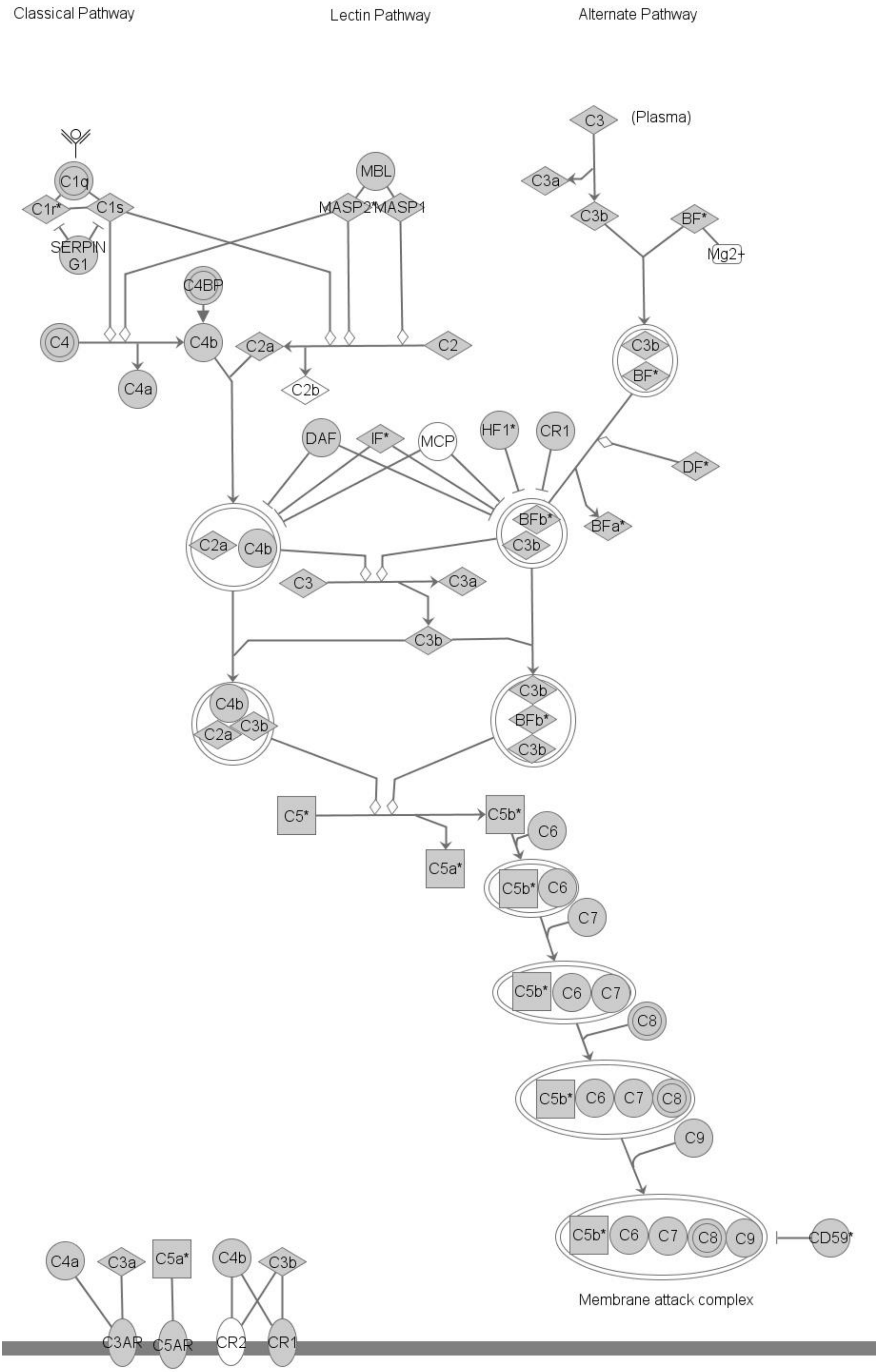

Classical Pathway
Lectin Pathway
Alternate Pathway
C3
(Plasma)
C3a
C1q
MBL
C1r*
C1s
MASP2*
MASP1
C3b
BF*
Mg2+
SERPIN G1
C4BP
C4
C4b
C2a
C2
C4a
C2b
C3b
BF*
DAF
IF*
MCP
HF1*
CR1
DF*
BFb*
C3b
BFa*
C2a
C4b
C3
C3a
C3b
C4b
C2a
C3b
C3b
BFb*
C3b
C5*
C5b*
C6
C5a*
C5b*
C6
C7
C5b*
C6
C7
C8
C5b*
C6
C7
C8
C9
C5b*
C6
C7
C8
C9
CD59*
Membrane attack complex
C4a
C3a
C5a*
C4b
C3b
C3AR
C5AR
CR2
CR1
