# Supplementary material for: Deep sequencing for de novo construction of a marine fish (Sparus aurata) transcriptome database with a large coverage of protein-coding transcripts
Source: BMC Genomics. 2013 Mar 15;14:178. doi: 10.1186/1471-2164-14-178 (PMC3606596; doi:10.1186/1471-2164-14-178)

Pipeline steps:

1) BLASTx search to a refseq protein database (in our case those employed during the functional analysis and annotation) to obtain the XML BLAST output with the information about all the hits for each query. You can do this considering or not a threshold of common similarity based on the HSP.

*Comments: In our case we first run the pipeline with a threshold of HSP > 80 (the query and the subject are similar in both sequence and size) from which we detected 11596 sequences with apparent frameshift and later we run a second analysis with which we detected 7765 additional sequences with apparent frameshifts. A summary of the results of each one or these two analyses is available within this Excel document.*

2) Parse the XML output to search sequences with two or more HSPs with distinct frames
then extract the queries with detected frameshift and create a sub-database. Do the same with the refseq protein subjects of the queries to correct.

3) First iteration: use HMMER and the protein subdatabase obtained in step 2 to perform a search to a database of HMMs

*Comments: In our case we used the PFAM and the GyDB profile DBs.*

4) First iteration: parse the HMMER output to see which proteins have an already existent HMM describing their consensus. Select these HMMs and save them in independent files (i.e. a file per HMM). Then take the subdatabase with the cDNAs with detected frameshifting and save in independent files those whose refseq proteins have HMMs.

5) First iteration: Correct all the cDNAs with HMMs by comparing them “file-to-file” and adding nucleotide insertions or deletions where corresponds, using HMM-FRAME.

6) Take the CDNAs of which a HMM was not detected for their refseq proteins (uncorrected cDNAs) and the corrected cDNAs and join both of them in a single file to perform a second HMM-FRAME iteration as described in 7.

7) Second iteration: extract from the refseq database the protein sequences corresponding with the “n” best hits for each query (two or more sequences the large number the better) save these sequences in independent files then creates a multiple alignment for each set of protein counterparts with CLUSTALW and next an HMM based on this alignment.

8) Second iteration: Correct all the cDNAs with specifically created HMMs by comparing them “file-to-file” and adding nucleotide insertions or deletions where corresponds, using HMM-FRAME

*Comments: In doing so additional corrections undetected in the first iteration performed with the sequences already corrected can be detected and corrected.*

9) Do the same than step 6 with the corrected and uncorrected sequences resulted from this iteration.

10) Third iteration: to correct sequences belonging to highly divergent protein families or detecting only one hit in the refseq protein database, extract the identified protein subject duplicate such a sequence and save it and its duplicate in a file. Then create an alignment and a “low accuracy” HMM profile based on this alignment

11) Third iteration: run HMM-FRAME to compare the cDNAs with the “low accuracy” HMMs “file-to-file” and adding “N” (any nucleotide) insertions or deletions where corresponds, using HMM-FRAME and an small script which substitute the nucleotide insertion suggested by HMM-FRAME by a “N”.

*Comments: We make this last implementation (change “N” by the HMM-FRAME nucleotide insertions) because the so called low accuracy HMMs created are not a real informative consensus, but just the sequence of one protein duplicated to have an HMM with which to apply the correction mechanisms of HMM-frame. By this reason and in absence of more information we prefer to add a “N“ in these positions where the tool inserts a nucleotide.*


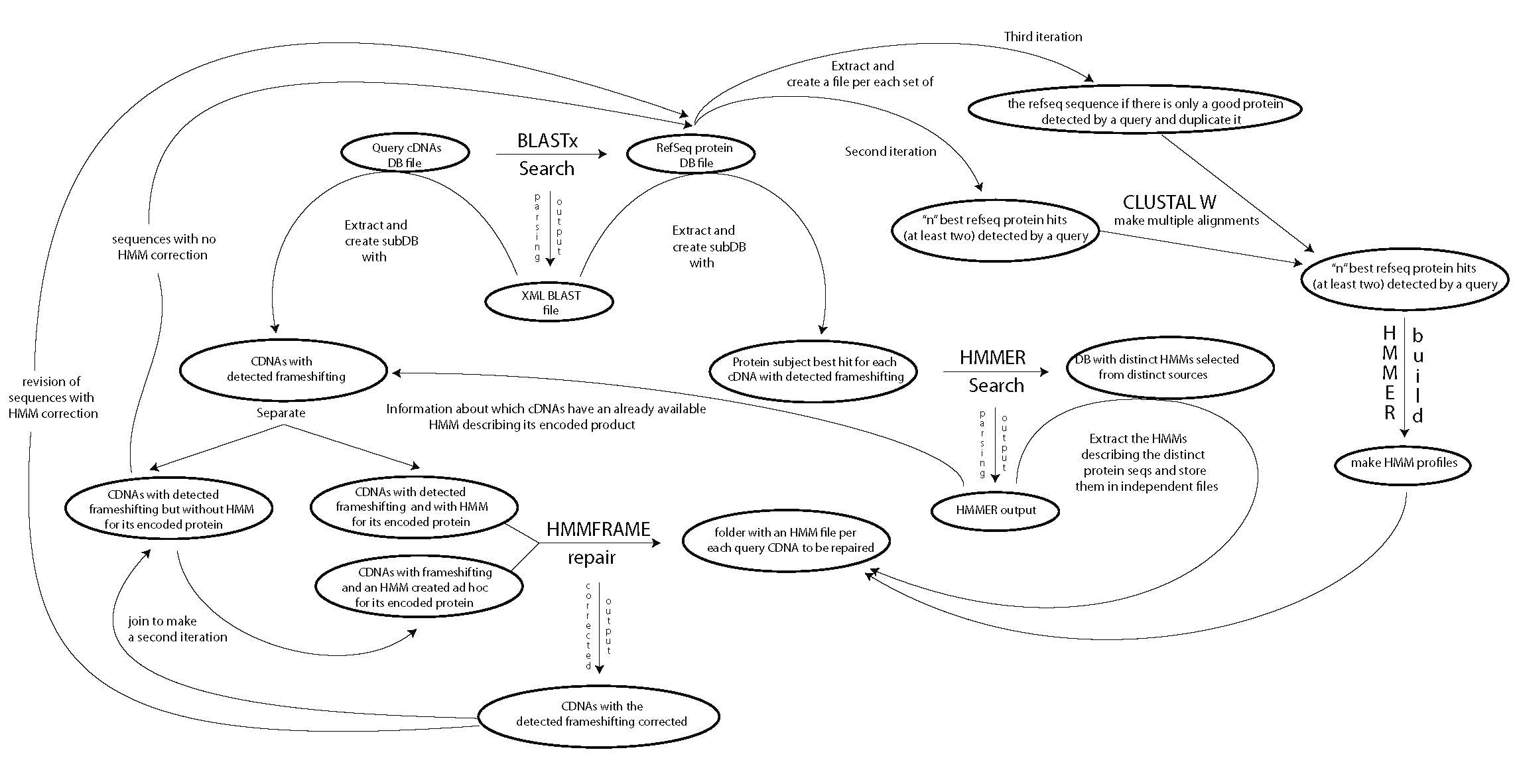

Supplement: Additional file 3 — Post-processing data and pipeline for frameshift correction. Zip file contains a Word document with an explanation of the distinct steps of the frameshifts processing pipeline accompanied by a graphical description of the pipeline schematics, and an Excel document reporting the frameshift corrections. [file 1471-2164-14-178-S3.zip › frameshift-pipeline.docx]
